# Carnauba wax-based edible coatings retain quality enhancement of orange (*Citrus sinensis* cv. Moro) fruits during storage

**DOI:** 10.1038/s41598-024-54556-1

**Published:** 2024-02-19

**Authors:** Mehrdad Babarabie, Ali Salehi Sardoei, Babak Jamali, Mehrnaz Hatami

**Affiliations:** 1 Department of Agriculture, Minab Higher Education Complex, University of Hormozgan, Bandar Abbas, Iran; 2 Horticultural and Crops Research Department Southern Kerman Agricultural and Natural Resources Research and Education Center, AREEO, Jiroft, Iran; 3 Department of Medicinal Plants, Faculty of Agriculture and Natural Resources, Arak University, Arak, 38156-8-8349 Iran

**Keywords:** Orange fruit, Coatings, Carnauba wax, Storage, Postharvest, Physiology, Plant sciences

## Abstract

Fruit coatings serve a dual purpose in preserving the quality of fruits. Not only do they act as a barrier against water evaporation and fungal infiltration, but they also enhance the fruit’s visual appeal in the market. Yet, their influence on the fruit’s quality components, which play a crucial role in determining its nutritional value, taste, and overall flavor, has remained relatively unexplored. This study aimed to evaluate the effects of carnauba wax coating on the quality of Moro oranges during storage. The selected fruits were meticulously chosen for uniformity in size. The experiment involved applying carnauba wax, a commonly used type among local producers, at four different concentrations: 0%, 0.5%, 1%, and 1.5%. These treatments were applied during various storage periods, including immediately after fruits were harvested and after 40 and 80 days. Following the application of these treatments, the oranges were stored in a controlled environment (morgue) at a temperature of 4 ± 1 °C. Subsequently, several physicochemical parameters of both the fruit flesh and skin were examined. The results unveiled a decline in the overall ascorbic acid content of the fruits. In terms of phenol content, a general decreasing trend was observed after harvesting. At each sampling interval during storage, the phenol content in uncoated fruits consistently exceeded that of their waxed counterparts. Significant reduction in fruit weight was observed throughout the storage period. Both vitamin C and total acidity levels in the fruit exhibited decreases during the storage period. As time passed, fruit firmness gradually declined, while fruit decay increased during the 40- and 80-day storage periods for untreated Moro oranges. The anthocyanin content showed an increasing trend. The study also unveiled a decline in the antioxidant capacity of citrus fruits during storage. Strong significant positive correlations were observed between total phenol content and key parameters, such as antioxidant activity (0.941^**^), MDA (0.364^*^), vitamin C content, and total carbohydrate content (0.475^**^). Skin radiance showed a perfect correlation with chroma and hue (1.000^**^). Principal component analysis revealed that the first principal component accounted for 34.27% of the total variance, out of a total of five principal components that explained 77.14% of the variance. Through cluster analysis, the variables were categorized into three distinct groups; one associated with weight loss and another with ion leakage. Considering these findings, carnauba wax-based coating emerges as a promising solution for preserving Moro oranges. It effectively mitigates fruit weight loss and helps maintain fruit firmness during storage, making it a valuable tool for fruit preservation.

## Introduction

Mazandaran province holds the prestigious rank of being the top producer of citrus fruits in Iran, with the largest cultivated area dedicated to citrus orchards. Out of the total citrus production in the country, which exceeds four million tons^1^, more than two million tons are attributed to this region. However, only a portion of this citrus yield enters the market directly, with the majority being stored for approximately 3 to 4 months, especially in conventional (cold) storage facilities. Less than optimal conditions in these storage facilities lead to high post-harvest losses each year. To mitigate these losses and enhance both the visual and nutritional quality of citrus fruits during postharvest storage, application of various coatings and proper storage conditions have been suggested by different authors^2–5^. Marcilla et al.^6^ studied different concentrations of wax coating on oranges and found that fruits treated with wax coating at 70% concentration exhibited the least weight reduction during storage.

While citrus fruits are generally classified as non-climacteric, the composition of these fruits can undergo changes depending on storage conditions, including temperature and storage duration^7^. Miranda et al.^8^ observed a decline in the vitamin C content of oranges during extended storage periods. This reduction in vitamin C content often coincides with a decrease in antioxidant capacity and overall fruit quality^9^. Furthermore, investigations by Klimezak and Malecka^10^ revealed significant fluctuations in the levels of polyphenols within citrus fruits, a variation that is influenced by storage temperature and duration. The alteration in total polyphenol levels during storage is contingent on the specific storage conditions^5^. In the case of blood oranges, there is typically an increase in the content of anthocyanins as the storage period progresses^11^. Palou et al.^12^ reported anthocyanins, flavonoids, and vitamin C levels increased during the storage of blood oranges. Higher antioxidant activity in stored blood oranges can be attributed to the synthesis of various phenolic compounds, including anthocyanins.

The difference in anthocyanin levels during the storage of blood oranges is correlated with the orange variety^13^. LoScalzo et al.^14^ demonstrated a remarkable increase in anthocyanin levels, up to fivefold, in the Tarocco oranges. In contrast, Moro oranges exhibited a more modest twofold increase, while the Sanguinello variety showed no change during storage. Additionally, the research revealed that the titratable acidity (TA) experienced a decline at the conclusion of the storage period across all varieties, except for Valencia oranges, where an increase was observed.

The storage temperature also significantly influences the sensory characteristics and flavor of fruits^15^. Manzano et al.^16^ investigated the impact of different temperature regimes on the aroma and taste of Valencia oranges during storage, it was found that higher temperatures had a detrimental effect on the distinctive citrus fruit flavor, leading to an increase in undesirable tastes. Furthermore, Obenland et al.^3^ reported that the aroma and taste of fruits underwent deterioration and developed unpleasant characteristics after four weeks of storage at varying temperatures.

According to studies, fruit coatings serve a dual purpose. They not only act as a protective barrier, preventing water evaporation and fungal decay, but also enhance the visual appeal of the fruits. Furthermore, considering the potential beneficial effects of these coatings on the production and preservation of valuable nutritional compounds, their use can play a role in promoting the overall health and well-being of the community. The impact of these coatings on the quality-associated parameters of fruits, which are crucial in determining their nutritional value, taste, and flavor, has received limited attention in previous literature. Hence, there is a pressing need for further research to better understand and optimize the application of coatings to preserve nutritional compounds for different fruit varieties. Moreover, it’s essential to acknowledge that quality standards for fruits have evolved in recent years. This underscores the necessity of staying current with shifting quality criteria to meet the evolving expectations of consumers and comply with regulatory requirements in the fruit industry. These days the quality parameters of a fruits (e.g., no/low levels of accumulation of toxic or harmful chemicals) are pivotal factors in determining import and export licenses for fruits. Consequently, it is imperative to place a heightened focus on the production of organic fruits that not only possess visual appeal but also boast high nutritional value. Most Iranian citrus producers rely on conventional storage methods for preserving fruits like Moro oranges. While there has been limited research on changes in these compounds during postharvest storage, this study aimed to investigate the impact of carnauba wax coating-a commonly used type among local producers-on the postharvest quality of Moro blood oranges.

## Materials and methods

### Fruit samples and treatments

Healthy, uniform Moro blood oranges (grafted on *Poncirus trifoliata* rootstock) with no signs of physical damage were harvested and collected from the orchard of the National Citrus Research Institute Research Station.

The fruits were divided into 12 groups, each containing 60 samples (three replications, 20 fruits each). Treatments included four different concentrations: 0%, 0.5%, 1%, and 1.5%. The coated fruits were stored for three months under normal storage conditions, i.e., 1 ± 4 degrees Celsius and relative humidity of 75% to 85%). At intervals of 0, 40, and 80 days, 15 fruits from each treatment were randomly selected and fruit quality parameters were measured.

### Physicochemical properties

To determine the reduction in fruit weight, three fruits from each replicate were individually labeled, weighed using a digital scale, and then placed in cold storage. At the end of the experimental period, the final weight of the fruits was determined again, and the weight loss^17^ was calculated using the following formula, expressed as a percentage:

$$ {\text{Fruit weight reduction }}\left( \% \right): \, \left[ {\left( {{\text{initial weight}} - {\text{final weight}}} \right)/{\text{initial weight}}} \right] \times {1}00. $$

To determine ion leakage, the electrolyte leakage index was utilized, following the method outlined by Sullivan and Ross^18^.

### Fruit decay Incidence

The percentage of fruit decay incidence is determined using the following formula^19^:

$$ {\text{Fruit Rot Incidence}} = \left( {{\text{n}}/{\text{N}}} \right) \times {1}00 $$

In this equation, N signifies the total count of fruits placed in storage, and n is the number of rotten fruits. Each fruit is individually harvested and sealed in a freezer bag to prevent contact with other fruits. Subsequently, these bags are tightly sealed and stored in a cold storage facility at a temperature of 4 ± 1 degrees Celsius.

### Fruit firmness

The fruit texture firmness was determined using a Penetrometer (Zwick, Germany)^17^.

### Vitamin C (ascorbic acid)

The concentration of vitamin C was determined using the titration method with 2,6-dichlorophenolindophenol (DIP) as described by^20^ with little modification]. Initially, 15 ml metaphosphoric acid (3%) was added to 5 ml of orange juice. The mixture was titrated with DIP containing sodium bicarbonate until a pink color appeared. The amount of vitamin C was calculated and expressed in mg per 100 ml fruit juice.

### Total phenol content

Total phenol content was determined utilizing the Folin-Ciocalteu method^21^. This entailed adding 470 μl of distilled water to 30 μl of fruit juice, followed by the addition of 2.5 ml of 10% Folin reagent. After a 3-min interval, 2 ml of 5.7% sodium carbonate were introduced. Following a 1.5-h incubation at room temperature, absorbance at a wavelength of 765 nm was recorded using a spectrophotometer. The results were expressed as mg of gallic acid equivalents per 100 ml fruit juice (mg GAE/100 g).

### Total antioxidant activity

The total antioxidant activity was determined using the DPPH (2,2-diphenyl-picryl-hydrazyl) radical degradation method. Specifically, 10 μL of fruit juice was combined with 4 mL of distilled water in test tubes, followed by the addition of 1 mL of a 250 μM DPPH solution. Subsequently, the test tubes were left undisturbed in darkness for a 30-min incubation period. Afterward, the absorbance was measured at 517 nm using a spectrophotometer^21^. The antioxidant activity was then calculated as the percentage of inhibition relative to the control, employing the following formula:

$$ {\text{Antioxidant}}\% = \left( {{\text{A blank}} - {\text{A sample}}/{\text{A blank}}} \right) \times {1}00 $$

where A blank is the absorbance of the control reaction, and A sample is the absorbance of the test compound in the sample.

### Total sugars

The total sugar content was determined using the Anthrone method. In this procedure, 200 μl of concentrated alcoholic extract was combined with 3 ml of Anthrone reagent and placed in a boiling water bath for 20 min. After cooling, the absorbance at 620 nm was measured for each treatment. The total sugar content of each sample was quantified by comparing it to a standard curve^22^.

### Titration analysis (TA)

Titration was carried out with 0.1 N NaOH using phenolphthalein as an indicator, and TA was expressed as grams of citric acid per 100 g of fruit juice according to the method discussed by^23^ with little modification].

### Anthocyanins

Total anthocyanins were measured spectrophotometrically using pH differential method with two buffer systems^24^: potassium chloride buffer, pH 1.0 (0.025 M) and sodium acetate buffer, pH 4.5 (0.4 M). Briefly, 0.4 ml of juice was mixed with 3.6 ml of corresponding buffers and read against water as blank at 510 and 700 nm. Absorbance (A) was calculated as

$$ {\text{A}} = \left( {{\text{A515}} - {\text{A7}}00} \right){\text{ pH 1}}.0 - \left( {{\text{A51}}0 - {\text{A7}}00} \right){\text{ pH 4}}.{5} $$

Then total anthocyanins content was calculated using the equation: Anthocyanin (mg · ml-1) = (A × MW × DF)/(ε × 1) Where A is the absorbance of the diluted sample and DF is the dilution factor. MW and in this formula correspond to the predominant anthocyanin in the sample. The pigment content was calculated as pelargonidin-3-glucoside, where MW = 433.38 and = 22.400.

### Malondialdehyde content

Malondialdehyde (MDA) content was assessed following the thiobarbituric acid (TBA) reaction, as originally outlined by Campos et al.^25^, with minor adjustments. Initially, 200 ml fruit juice were homogenized in 2 mL of 0.1% trichloroacetic acid and subjected to centrifugation at 10,000 × g for 15 min. Next, 1 mL of the resulting supernatant was combined with 2.5 mL of 0.5% thiobarbituric acid in 20% trichloroacetic acid and subjected to incubation in hot water at 95 °C for 30 min. The reaction was promptly terminated by cooling the mixture on ice, followed by centrifugation at 10,000 × g for 30 min. The absorbance was measured at 532 and 600 nm. The concentration of MDA was determined by subtracting the nonspecific absorption at 600 nm from the absorption at 532 nm, using an absorbance coefficient of extinction (155 mM^−1^ cm^−1^).

### Sensory assessment

Towards the conclusion of the storage period, a sensory evaluation was carried out with the participation of nine randomly selected assessors (untrained consumers of ages 18–45 years among students and employees). These assessors provided ratings on a scale from 1 to 10, considering various attributes including visual appearance (skin and flesh characteristics), aroma, taste, sweetness, tartness, bitterness, and overall acceptability of the fruit. A 10-point hedonic scale ranging from 10 for “like extremely” to 1 for “dislike extremely” was used to conduct the sensory test^26^.

### Statistical analysis

The experiment was carried out in a factorial design within a completely randomized setup, evaluating two factors: coating concentration and the storage duration. Data were analyzed by SAS, and means were compared using Duncan’s multiple range tests at the 5% probability level. SPSS was used for correlation coefficients and cluster analysis.

### Permission to collect fruit samples

The permission to collect *Citrus sinensis* cv. Moro fruits was acquired from Agricultural and Natural Resources Ministry of Iran.

### Statement on experimental research and field studies on plants

The plants (*Citrus sinensis* cv. Moro) sampled comply with relevant institutional, national, and international guidelines and domestic legislation of Iran.

## Results

### Total phenol content in fruit flesh

The effect of carnauba coating treatment and storage time on the total phenol content of fruit juice was significant at the 5% and 1% probability levels, respectively, individually, but their interaction effect was non-significant (Table 1). In Table 1, descriptive statistics, including Mean ± SE (0.51 ± 0.025) and standard deviations (0.151), minimum (0.112) and maximum values (0.722), the range of variations (0.61), for various parameters, have been summarized.

**Table 1:** Descriptive statistical analysis and analysis of variance for total phenol content of Moro oranges during storage

Traits	Mean ± Standard error	Standard variation	Range	Minimum	Maximum
Total phenol	0.51 ± 0.025	0.151	0.61	0.112	0.722

S.O.V	Carnauba wax	Storage time	Carnauba wax * Storage time	Cv	
df	3	2	6	–	
Total phenol	0.041*	9.485**	0.096^ns^	20.98	

* and ** significant at 5% and 1% levels, respectively. ns: non-significant.

**Figure 1:**
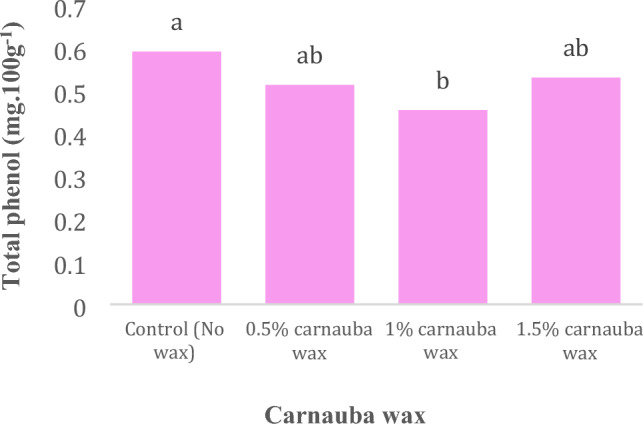
Effect of coating on total phenol content in Moro orange fruit juice. Different letters above the bars indicate they are significantly different by Duncan’s multiple at *P* < 0.05.

Notably, there were no significant differences observed among various levels of carnauba treatment, as depicted in Fig. 1. A comparison of mean values revealed that the highest and lowest total phenol content were observed at control (No wax) and 1% of storage, respectively (Fig. 1).

### Skin color, chroma, and hue

The effect of carnauba coating treatment on the skin color, chroma, hue and ion leakage of fruit juice was significant at the 1% probability levels, respectively, individually, but carnauba coating treatment and their interaction effect was non significant (Table 2). The highest percentages of phenotypic variation were exhibited by ion leakage (288), and characteristics related to skin radiance, chroma, and hue (28.29) (Table 2).

**Table 2:** Descriptive statistical analysis and analysis of variance for ion leakage, skin radiance (L*), Chroma and Hue of Moro oranges during storage

Traits	Mean ± Standard Error	Standard variation	Range	Minimum	Maximum
Ion leakage	132.26 ± 9.75	57.706	288	61	349
Skin radiance (L*)	38.2 ± 1.262	7.469	28.29	26.24	54.53
Chroma	43.4 ± 1.262	7.469	28.29	31.45	59.74
Hue	47.6 ± 1.262	7.469	28.29	35.57	63.86

S.O.V	Carnauba wax	Storage time	Carnauba wax*Storage time	Cv	
df	3	2	6	–	
Ion leakage	5496.4^ns^	562,584.4**	1837.5^ns^	23.67	
Skin radiance (L*)	63.45^ns^	48,561.5**	13.38^ns^	18.09	
Chroma	63.45^ns^	62,700.3**	0.626^ns^	10.21	
Hue	63.45^ns^	75,158.5**	13.38^ns^	16.65	

* and ** significant at 5% and 1% levels, respectively. ns: non-significan.

**Figure 2:**
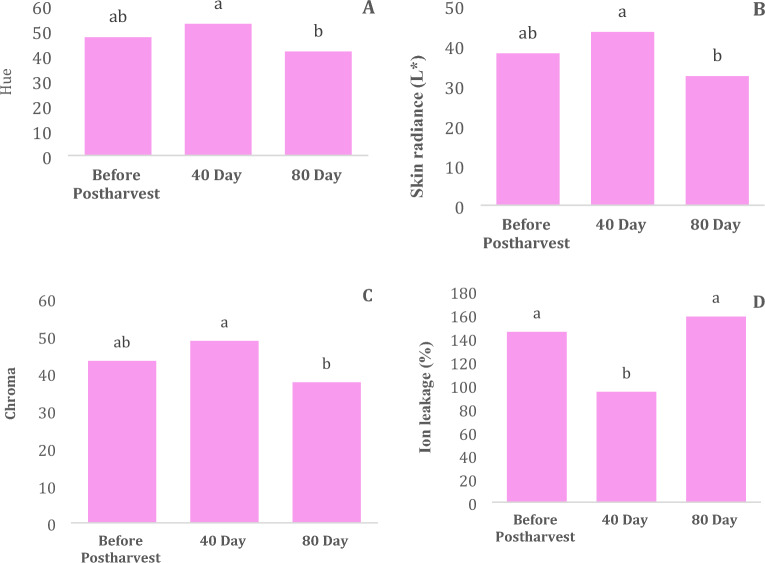
Effect of Coating on hue (**A**), skin radiance (**B**), chroma (**C**), and ion leakage (**D**) in Moro orange fruit. Different letters above the bars indicate they are significantly different by Duncan’s multiple at *P* < 0.05.

The results of the color analysis for both wax-coated and control (uncoated) oranges during the 90-day storage period are presented in Fig. 2A–C. According to Table 2, the effect of storage time on skin color, chroma, and hue was significant, whereas the carnauba treatment and its interaction with storage time did not yield significant effects. A comparison of mean values revealed that the highest and lowest skin color, chroma, and hue were observed at day 40 and day 80 of storage, respectively (Fig. 2A–C).

### Ion leakage

Based on the results of the analysis of variance, neither the carnauba treatment nor its interaction with storage time had a significant impact on ion leakage. However, when considered individually, storage time exhibited a significant effect on ion leakage, with the highest and lowest levels observed on days 80 and 40 of storage, respectively (Fig. 2D).

### The panel test, flavor, and overall acceptability

The effect of carnauba treatment, storage time, and their interaction on the panel test, flavor, and overall acceptability of oranges were significant (Table 3). The highest percentages of phenotypic variation were exhibited by panel test (2.42), and characteristics related to flavour and taste (2.3), little acceptance (2) (Table 3).

**Table 3:** Descriptive statistical analysis and analysis of variance for panel test, flavour and taste and little acceptance of Moro oranges during storage.

Traits	Mean ± Standard Error	Standard variation	Range	Minimum	Maximum
Panel test	1.39 ± 0.086	0.508	2.42	0.58	3
Flavour and Taste	1.29 ± 0.087	0.517	2.3	0.5	2.8
Little acceptance	1.43 ± 0.088	0.520	2	1	3

S.O.V	Carnauba wax	Storage time	Carnauba wax * Storage time	Cv	
df	3	2	6	–	
Panel test	0.481*	63.13**	1.5*	26.51	
Flavour and Taste	0.492*	55.93**	0.51*	27.67	
Little acceptance	0.478*	66.7**	1.26*	27.61	

* and ** significant at 5% and 1% levels, respectively. ns: non-significan.

As illustrated in Table 4, the highest scores for the panel test and overall acceptability were associated with the carnauba treatment (0.5%) on day 40 of storage. Conversely, the lowest scores for these two parameters were observed in the control group as well as with a carnauba concentration of 1.0% at the beginning of storage and on day 40 of orange fruit storage. The highest and lowest taste and flavor scores, respectively, were documented at the carnauba concentration (0.5%) on day 40 of storage and the carnauba concentration (1.0%) on day 80 of storage (Table 4).

**Table 4:** The interaction effect of carnauba wax coating and storage time on The panel test, flavor, and overall acceptability.

Carnauba Wax Coating	Storage time (Days)	Little acceptance	Flavour and taste	Panel test
No wax	0 (Before Postharvest)	1c	1cd	1c
0.5% carnauba wax	0 (Before Postharvest)	1.44abc	1.16bcd	1.62bc
1% carnauba wax	0 (Before Postharvest)	2.08a	1.91a	2ab
1.5% carnauba wax	0 (Before Postharvest)	1c	1cd	1c
No wax + 40 Day	40	1.55abc	1.46abc	1.54bc
0.5% carnauba wax	40	2.15a	2.1a	2.3a
1% carnauba wax	40	1c	1cd	1c
1.5% carnauba wax	40	1.55abc	1.05bcd	1.24c
No wax	80	1.78ab	1.73ab	1.48bc
0.5% carnauba wax	80	1.11bc	1.11bcd	1.22c
1% carnauba wax	80	1.26bc	0.75d	1.19c
1.5% carnauba wax	80	1.43abc	1.65abc	1.37bc

### Total sugars

The total sugar content in orange fruits was significantly influenced by the carnauba wax treatment, storage duration, and their interaction effects (Table 5). In Table 5, descriptive statistics, including Mean ± SE (14.9 ± 0.807) and standard variation (4.778), minimum (0.57) and maximum values (17.91), the range of variations (17.34), for various parameters, have been summarized.

**Table 5:** Descriptive statistical analysis and analysis of variance for total sugar of Moro oranges during storage.

Trait	Mean ± Standard Error	Standard variation	Range	Minimum	Maximum
Total sugar	14.9 ± 0.807	4.778	17.34	0.57	17.91

S.O.V	Carnauba wax	Storage time	Carnauba wax * Storage time	Cv	
*d*f	3	2	6	–	
Total sugar	55.19**	7448.7**	195.1**	10.32	

* and ** significant at 5% and 1% levels, respectively. ns: non-significan.

**Figure 3:**
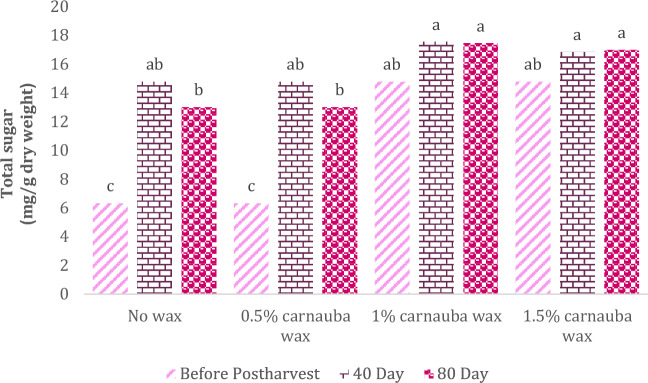
The interaction effect of carnauba wax coating and storage time on total sugars. Different letters above the bars indicate they are significantly different by Duncan’s multiple at *P* < 0.05.

As depicted in Fig. 3, the highest total sugar content was observed in the following treatments: no carnauba wax treatment on both day 40 and day 80 of storage, carnauba wax treatment (1%) on day 40 of storage, and carnauba wax coating (1.5%) 80 days after storage. There were no statistically significant differences among these conditions.

### TA

The statistical analysis of the data revealed significant effects of carnauba wax treatment and storage duration on fruit acidity levels (Table 6). In Table 6, descriptive statistics, including Mean ± SE (3 ± 0.249) and standard variation (1.47), minimum (1.29) and maximum values (8.3), the range of variations (7.01), for various parameters, have been summarized.

**Table 6:** Descriptive statistical analysis and Analysis of variance for TA of Moro oranges during storage.

Trait	Mean ± Standard Error	Standard variation	Range	Minimum	Maximum
TA	3 ± 0.249	1.47	7.01	1.29	8.3

S.O.V	Carnauba wax	Storage time	Carnauba wax * Storage time	Cv	
df	3	2	6	–	
TA	5.15**	296.1**	0.018*	18.86	

* and ** significant at 5% and 1% levels, respectively. ns: non-significan.

**Figure 4:**
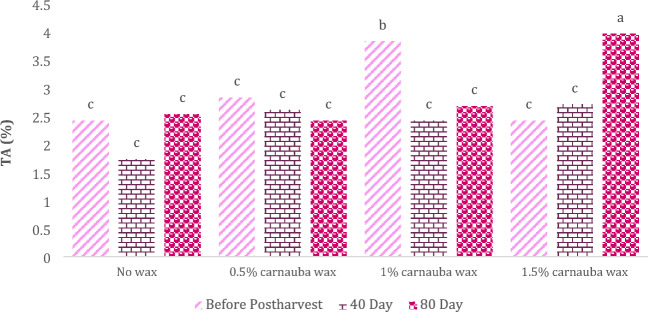
The interaction effect of carnauba wax coating and storage time on total acids. Different letters above the bars indicate they are significantly different by Duncan’s multiple at *P* < 0.05.

As depicted in Fig. 4, the highest fruit acidity levels in oranges were observed in fruits treated with a 1.5% carnauba wax concentration, after 80 days of storage, followed by those treated with a 1% carnauba wax concentration after 40 days of storage. Other carnauba wax concentrations did not show significant differences. At the conclusion of the 80-day storage period, the lowest total acidity (TA) was recorded in fruits treated with carnauba wax (0.5%).

### Vitamin C

As shown in Table 7, the impact of carnauba wax treatment on vitamin C content was non-significant. In Table 7, descriptive statistics, including Mean ± SE (0.84 ± 0.056) and standard variation (0.336), minimum (0.25) and maximum values (1.38), the range of variations (1.13), for various parameters, have been summarized.

**Table 7:** Descriptive statistical analysis and analysis of variance for Vitamin C of Moro oranges during storage.

Trait	Mean ± Standard Error	Standard variation	Range	Minimum	Maximum
Vitamin C	0.84 ± 0.056	0.336	1.13	0.25	1.38

S.O.V	Carnauba wax	Storage time	Carnauba wax * Storage time	Cv	
df	3	2	6	–	
Vitamin C	0.180^ns^	24.3**	0.006*	26.92	

* and ** significant at 5% and 1% levels, respectively. ns: non-significan.

**Figure 5:**
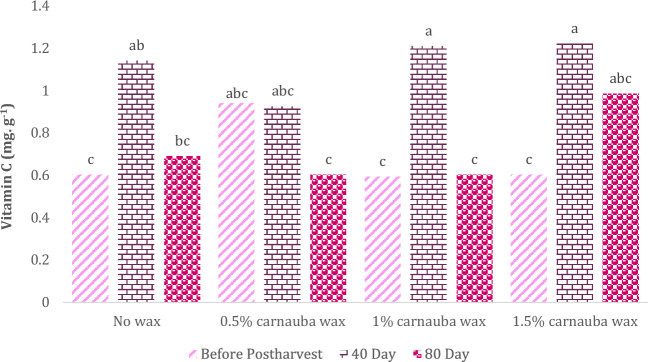
The interaction effect of carnauba wax coating and storage time on vitamin C content in fruits. Different letters above the bars indicate they are significantly different by Duncan’s multiple at *P* < 0.05.

However, the storage duration and its interaction with carnauba treatment influenced this parameter significantly. As illustrated in Fig. 5, the highest vitamin C concentration was observed in fruits treated with 1% or 1.5% carnauba wax concentrations during both the 40 and 80 days of storage, with no significant difference between them. On the contrary, the lowest levels of this parameter were recorded in the control group and at the beginning of the storage period.

### Weight loss

The weight loss in orange fruits was significantly influenced by the carnauba wax treatment, storage duration, and their interaction effects (Table 8). In Table 8, descriptive statistics, including Mean ± SE (313.1 ± 7.535) and standard variation (44.579), minimum (221.27) and maximum values (406.9), the range of variations (185.63), for various parameters, have been summarized.

**Table 8:** Descriptive statistical analysis and analysis of variance for weight loss of Moro oranges during storage.

Trait	Mean ± Standard Error	Standard variation	Range	Minimum	Maximum
Weight loss	313.1 ± 7.535	44.579	185.63	221.27	406.9

S.O.V	Carnauba wax	Storage time	Carnauba wax * Storage time	Cv	
df	3	2	6	–	
Weight loss	3640.6**	3,256,576.7**	282.3**	9.16	

* and ** significant at 5% and 1% levels, respectively. ns: non-significant.

**Figure 6:**
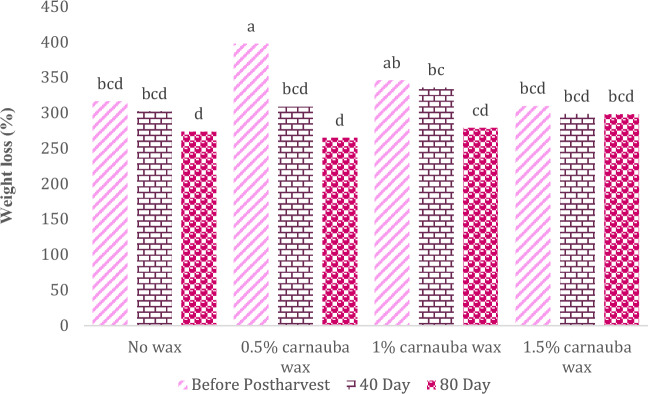
The interaction effect of carnauba wax coating and storage time on fruit weight reduction. Different letters above the bars indicate they are significantly different by Duncan’s multiple at *P* < 0.05.

Figure 6 also shows that the highest and lowest rates of weight loss were observed in samples treated with carnauba wax at 0.5% concentration after 80 and 40 days of storage, respectively.

### Fruit peel firmness

The statistical analysis of the data revealed significant effects of carnauba wax treatment and storage duration on firmness (Table 9). In Table 9, descriptive statistics, including Mean ± SE (2.72 ± 0.097) and standard variation (0.574), minimum (1.91) and maximum values (3.81), the range of variations (1.9), for various parameters, have been summarized.

**Table 9:** Descriptive statistical analysis and analysis of variance for firmness of Moro oranges during storage.

Trait	Mean ± Standard Error	Standard variation	Range	Minimum	Maximum
Firmness	2.72 ± 0.097	0.574	1.9	1.91	3.81

S.O.V	Carnauba wax	Storage time	Carnauba wax * Storage time	Cv	
df	3	2	6	–	
Firmness	0.693**	248.8**	0.086*	10.21	

* and ** significant at 5% and 1% levels, respectively. ns: non-significant.

**Figure 7:**
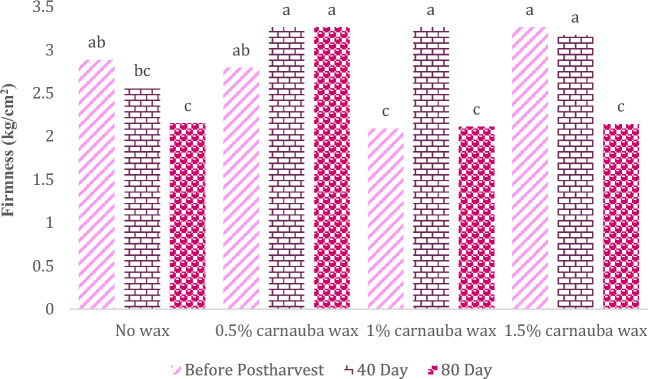
The interaction effect of carnauba wax coating and storage time on fruit peel firmness. Different letters above the bars indicate they are significantly different by Duncan’s multiple at *P* < 0.05.

The carnauba treatment, storage time, and their interaction influenced this parameter significantly. The highest level of firmness was observed in control and fruit samples treated with carnauba wax concentrations (0.5% and 1%.) after 40 days of storage (Fig. 7).

### Fruit decay

Carnauba treatment, storage time, and their interaction significantly influence fruit decay (Table 10). In Table 10, descriptive statistics, including Mean ± SE (0.369 ± 0.101) and standard variation (0.199), minimum (0) and maximum values (1.75), the range of variations (1.75), for various parameters, have been summarized.

**Table 10:** Descriptive statistical analysis and analysis of variance for decay of Moro oranges during storage.

Trait	Mean ± Standard Error	Standard variation	Range	Minimum	Maximum
Decay	0.369 ± 0.101	0.199	1.75	0	1.75

S.O.V	Carnauba wax	Storage time	Carnauba wax * Storage time	Cv	
df	3	2	6	–	
Decay	0.101**	11.67**	0.232**	19.32	

* and ** significant at 5% and 1% levels, respectively. ns: non-significant.

**Figure 8:**
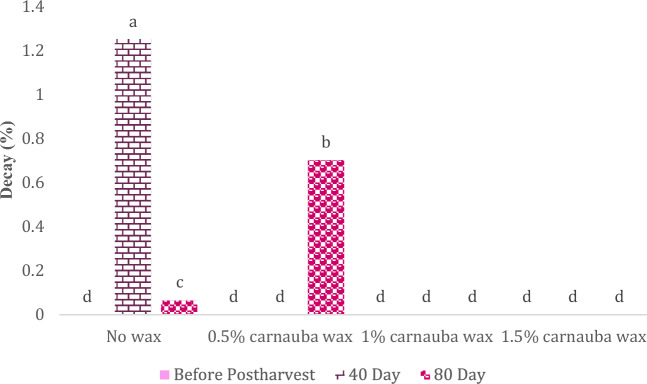
The interaction effect of carnauba wax coating and storage time on fruit decay. Different letters above the bars indicate they are significantly different by Duncan’s multiple at *P* < 0.05.

As illustrated in Table 11 and Fig. 8, the highest fruit decay rate in oranges was observed after 40 and 80 days of storage without carnauba treatment.

**Table 11:** The interaction effect of carnauba wax coating and storage time on fruit decay.

	Before postharvest	40	80 day
No wax	−	+	+
0.5% carnauba	−	−	+
1% carnauba wax	−	−	−
1.5% carnauba wax	−	−	−

### Malondialdehyde

Carnauba treatment, storage duration, and their interaction significantly affected the MDA content (Table 12). In Table 12, descriptive statistics, including Mean ± SE (0.63 ± 0.062) and standard variation (0.369), minimum (0.060) and maximum values (1.157), the range of variations (1.097), for various parameters, have been summarized.

**Table 12:** Descriptive statistical analysis and analysis of variance for MDA of Moro oranges during storage.

Trait	Mean ± Standard Error	Standard variation	Range	Minimum	Maximum
MDA	0.63 ± 0.062	0.369	1.097	0.060	1.157

S.O.V	Carnauba wax	Storage time	Carnauba wax * Storage time	Cv	
df	3	2	6	–	
MDA	0.308**	13.67**	0.012**	29.92	

* and ** significant at 5% and 1% levels, respectively. ns: non-significant.

**Figure 9:**
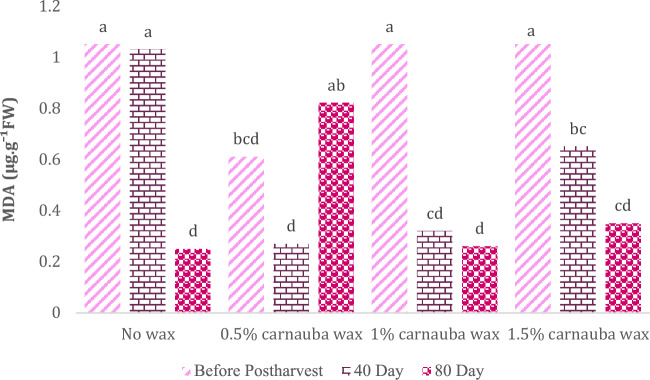
The interaction effect of carnauba wax coating and storage time on MDA level. Different letters above the bars indicate they are significantly different by Duncan’s multiple at *P* < 0.05.

The highest MDA concentration was observed in the control group after fruit harvest. There were no differences in MDA content when carnauba was applied at concentrations of 0.5%, 1%, and 1.5% on days 80, 40, and at the beginning of cold storage, respectively. A steady decrease in MDA content was observed as the storage period progressed, indicating a beneficial effect of the storage period on mitigating this characteristic (Fig. 9).

### Anthocyanin

Carnauba treatment, storage duration, and their interaction significantly affected this parameter (Table 13). In Table 13, descriptive statistics, including Mean ± SE (1.44 ± 0.071) and standard variation (0.424), minimum (0.421) and maximum values (2.396), the range of variations (1.975), for various parameters, have been summarized.

**Table 13:** Descriptive statistical analysis and analysis of variance for Anthocyanin of Moro oranges during storage.

Trait	Mean ± Standard Error	Standard variation	Range	Minimum	Maximum
Anthocyanin	1.44 ± 0.071	0.424	1.975	0.421	2.396

S.O.V	Carnauba wax	Storage time	Carnauba wax * Storage time	Cv	
df	3	2	6	–	
Anthocyanin	0.280*	70.23**	0.018*	23.83	

* and ** significant at 5% and 1% levels, respectively. ns: non-significant.

**Figure 10:**
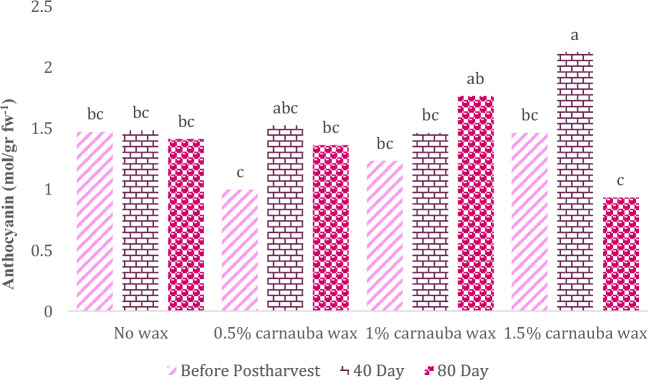
The interaction effect of carnauba wax coating and storage time on anthocyanin content in fruits. Different letters above the bars indicate they are significantly different by Duncan’s multiple at *P* < 0.05.

The highest and lowest levels of anthocyanin content in fruit were observed in the carnauba treatment (1.5%) on days 40 and 80 after cold storage, as illustrated in Fig. 10. As the storage period extended to 40 days, there was an increase in fruit anthocyanin levels.

### Antioxidant activity

This parameter was significantly influenced by Carnauba treatment, storage duration, and their interaction (Table 14). In Table 14, descriptive statistics, including Mean ± SE (0.38 ± 0.022) and standard variation (0.134), minimum (0.064) and maximum values (0.582), the range of variations (0.518), for various parameters, have been summarized.

**Table 14:** Descriptive statistical analysis and analysis of variance for antioxidant activity of Moro oranges during storage.

Trait	Mean ± Standard Error	Standard variation	Range	Minimum	Maximum
Antioxidant activity	0.38 ± 0.022	0.134	0.518	0.064	0.582

S.O.V	Carnauba wax	Storage time	Carnauba wax * Storage time	Cv	
df	3	2	6	–	
Antioxidant activity	0.096*	5.392**	0.096*	21.45	

* and ** significant at 5% and 1% levels, respectively. ns: non-significant.

**Figure 11:**
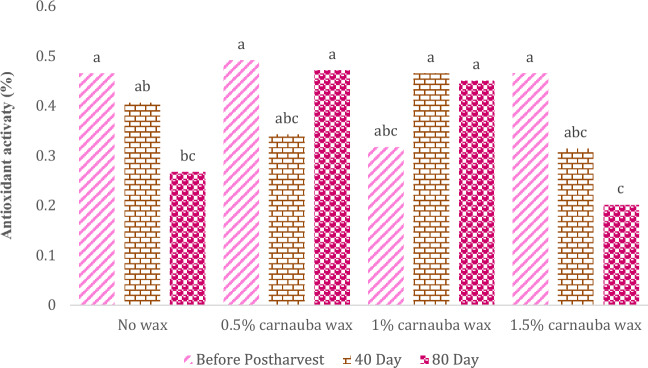
The interaction effect of carnauba wax coating and storage time on antioxidant activity of fruits. Different letters above the bars indicate they are significantly different by Duncan’s multiple at *P* < 0.05.

The peak antioxidant activity of fruits was observed in fruits treated with carnauba wax (0.5%) immediately after harvest. However, a general decline in antioxidant activity was observed when fruits were cold stored for longer periods, except for the carnauba treatment (1%), which showed an increase on days 40 and 80 (Fig. 11).

### Correlation coefficients for assessing relationships among characteristics

**Figure 12:**
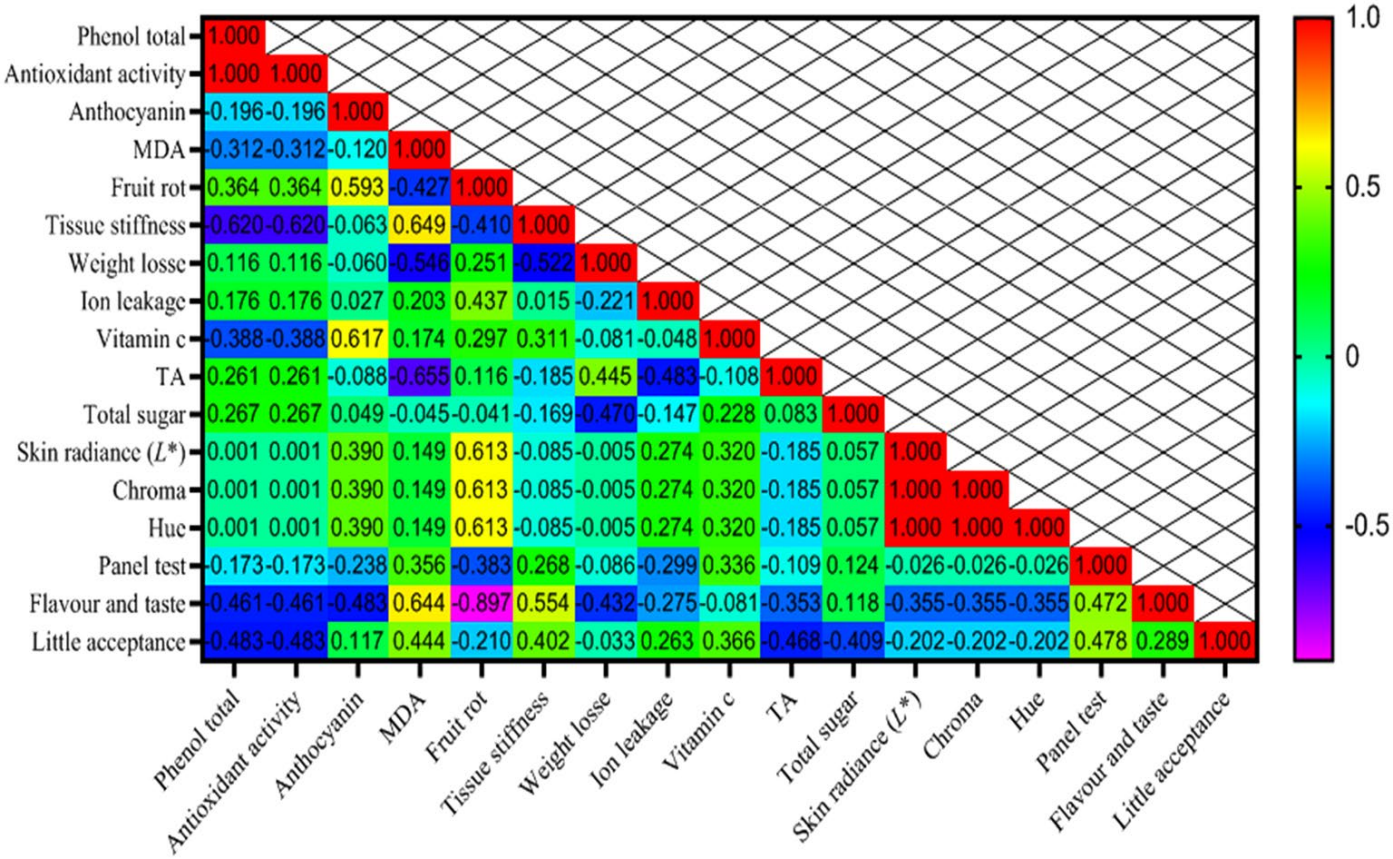
Heat map of relations among variables in correlation coefficient for Physicochemical indicators for 40 days.

**Figure 13:**
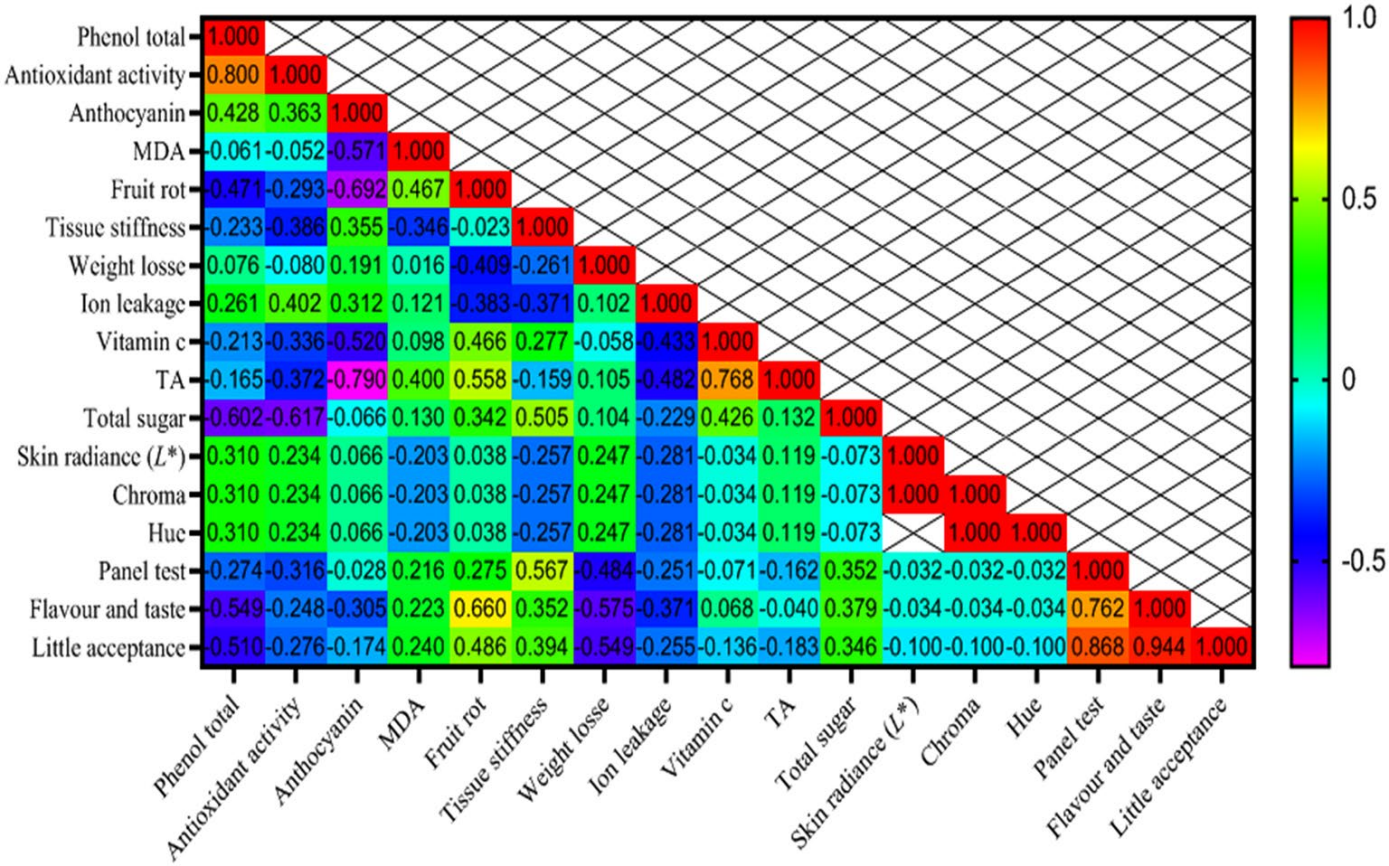
Heat map of relations among variables in correlation coefficient for Physicochemical indicators for 80 days.

**Figure 14:**
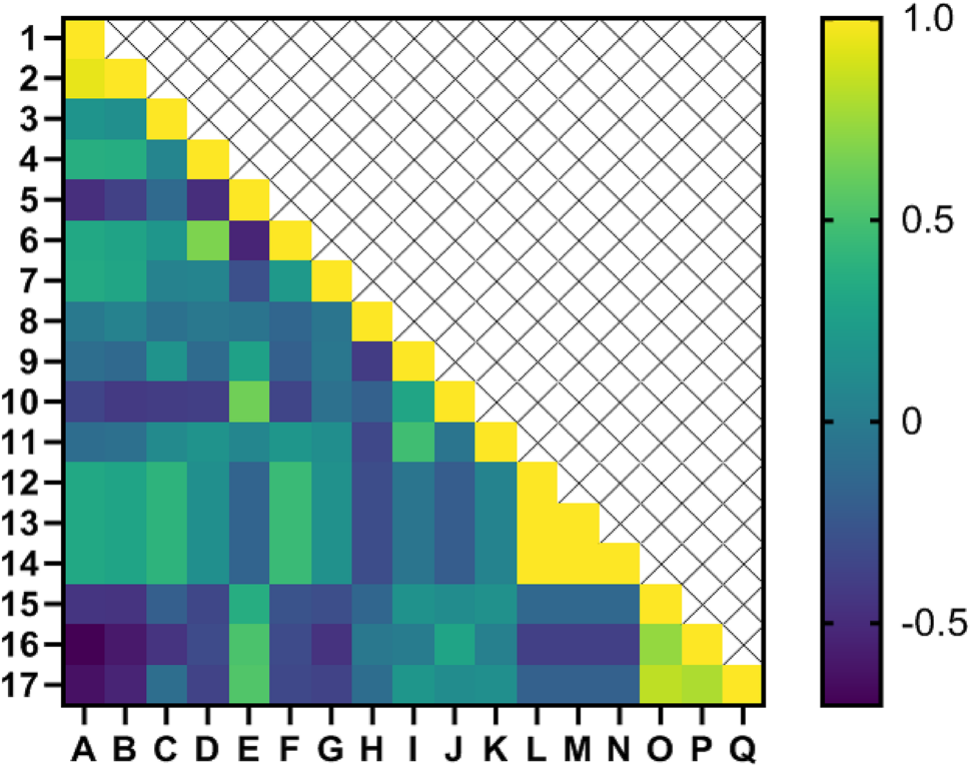
Heat map of relations among variables in correlation coefficient for Physicochemical indicators. # Y axis are respectively: 1—Total phenolic compounds, 2—Antioxidant, 3—Anthocyanin, 4—MDA, 5—Fruit decay rate, 6—Fruit peel firmness, 7—Weight reduction, 8—Ion leakage, 9—Vitamin C, 10—TA, 11—Total sugars, 12—Skin radiance (L*), 13—Chroma, 14—Hue, 15—Panel test, 16—Flavor and taste, 17—Little acceptance. # X axis are respectively: A—Total phenolic compounds, B—Antioxidant activity, C—Anthocyanin, D—MDA, E—Fruit decayrate, F—Fruit peel firmness, G—Weight reduction, H—Ion leakage, I—Vitamin C, J—TA, K—Total sugars, L—Skin radiance (L*), M—Chroma, N—Hue, O—Panel test, P—Flavor and taste, Q—Little acceptance.

The Pearson correlation coefficients illustrating the relationships between the 40, 80 days and (combined) days traits studied are shown in Fig. 12. Here are the findings:

- Total Phenolic compounds exhibited a robust positive correlation with Antioxidant activity (1.000**).
- Anthocyanin exhibited a moderately positive correlation with Vitamin c (0.617*) and Fruit rot (0.593*).
- MDA exhibited a moderately positive correlation with Flavour and taste (0.644*) and Fruit rot (0.649*).
- Fruit rot exhibited a moderately positive correlation with Skin radiance (L*), Chroma and Hue (0.613*).
- Tissue stiffness exhibited a moderately positive correlation with flavour and taste (0.554*).
- Panel test exhibited a moderately positive correlation with little acceptance (0.478*) and flavour and taste (0.472*) (Fig. 12).
- Total Phenolic compounds exhibited a robust positive correlation with Antioxidant activity (0.800**).
- Fruit rot exhibited a moderately positive correlation with little acceptance (0.486*), flavour and taste (0.660*), TA (0.558*) and vitamin c (0.466*).
- Tissue stiffness exhibited a moderately positive correlation with panel test (0.567*).
- Vitamin c exhibited a moderately positive correlation with total sugar (0.426*).
- Panel test exhibited a robust positive correlation with flavor and taste (0.762**).
- Flavor and taste exhibited a robust positive correlation with little acceptance (0.944**) (Fig. 13).
- Total Phenolic compounds exhibited a robust positive correlation with Antioxidant activity (0.941**) and a moderately positive correlation with MDA (0.364*).
- Antioxidant activity displayed a moderate positive correlation with MDA (0.349*).
- Anthocyanin content demonstrated a moderate positive correlation with Skin radiance (L*), Chroma, and Hue (0.395*).
- MDA manifested a strong positive correlation with fruit peel firmness (0.668**).
- Fruit decay rate showcased strong positive correlations with total acids (0.631**), Panel test (0.356*), Flavor and taste (0.513**), and Little acceptance (0.534**).
- Firmness featured a moderate positive correlation with Skin radiance (L*), Chroma, and Hue (0.451*).
- Vitamin C displayed a moderate positive correlation with Total Sugars (0.475**).
- Skin radiance demonstrated a perfect correlation with Chroma and Hue (1.000**).
- Chroma and Hue exhibited a perfect correlation with each other (1.000**).
- Panel test revealed a strong positive correlation with Flavor and taste (0.729**) and Little acceptance (0.833**).
- Flavor and taste showed a strong positive correlation with Little acceptance (0.791**) (Fig. 14).

### Cluster analysis

**Figure 15:**
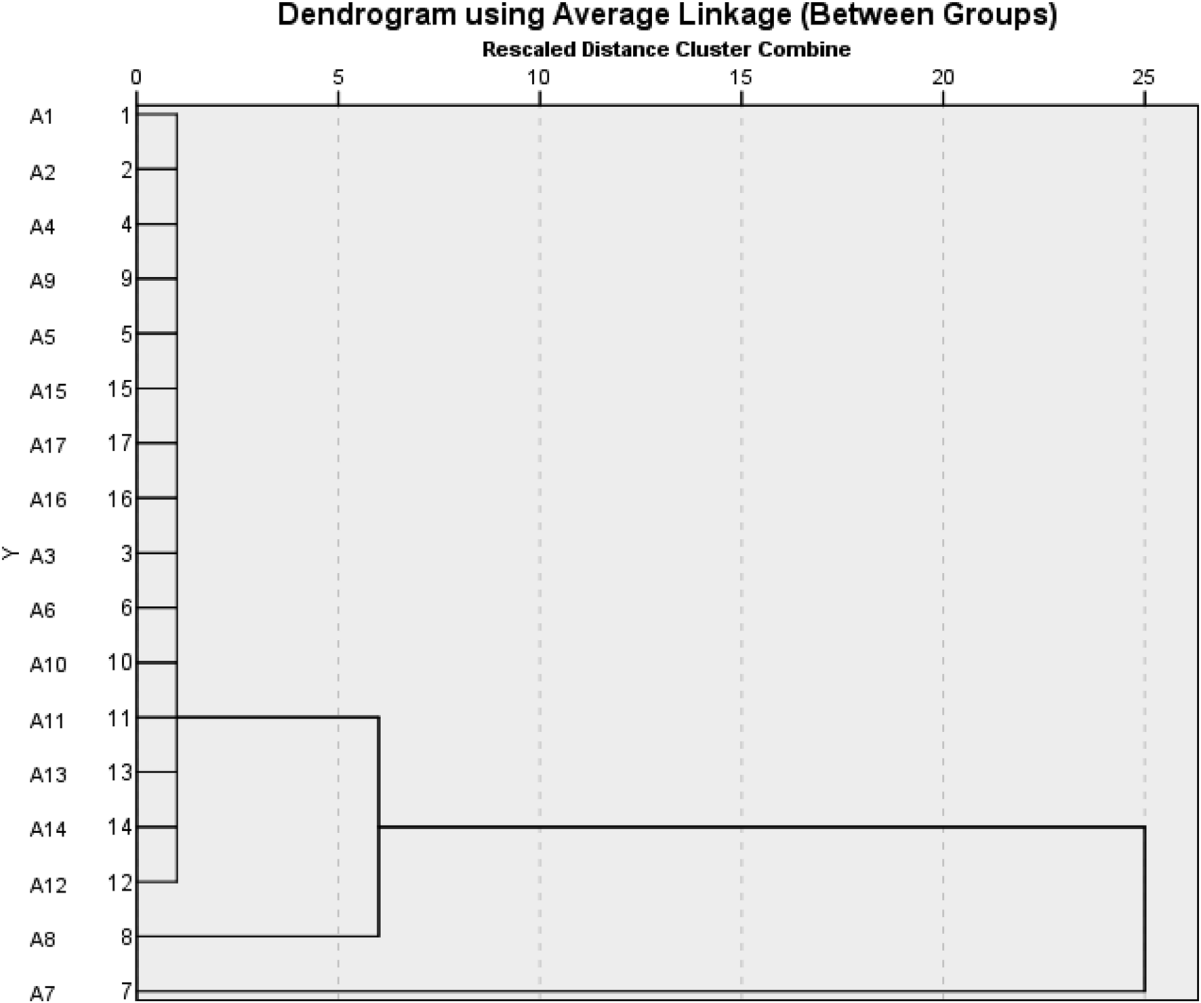
Cluster analysis of physicochemical characteristics of Moro Orange fruit during storage. 1—Total phenolic compounds, 2—Antioxidant, 3—Anthocyanin, 4—MDA, 5—Fruit decay rate, 6—Fruit peel firmness, 7—Weight reduction, 8—Ion leakage, 9—Vitamin C, 10—TA, 11—Total sugars, 12—Skin radiance (L*), 13—Chroma, 14—Hue, 15—Panel test, 16—Flavor and taste, 17—Little acceptance.

The cluster analysis dendrogram illustrates the segmentation of the 17 physicochemical parameters into three clusters (Fig. 15).

The first cluster included one parameter: the friut weight reduction. The second cluster included Ion leakage. The remaining characteristics were positioned in the third cluster.

## Discussion

Edible coatings are being considered as the packaging solution of the future, holding promise in minimizing postharvest losses attributed to moisture loss. These coatings consist of thin layers of food-grade biopolymers derived from proteins, polysaccharides, or lipids. They are directly applied to the product’s surface, seamlessly adhering to this surface as an integral part of the final product. These materials act as a protective shield against physical damage, microbial contamination, moisture loss, and nutritional oxidation^26^. Consequently, they play a pivotal role in preventing product spoilage, thereby elongating the product’s shelf life, preserving sensory quality, and ensuring safety^27^. What sets these coatings apart from traditional plastic packaging is their edibility, biodegradability, and the potential to partially or fully replace plastic alternatives^28^.

The utilization of carnauba wax as a coating on fruit surfaces serves a dual purpose—enhancing the visual appeal and safeguarding the fruit against decay. This can extend the fruit’s shelf life and mitigate post-harvest losses. Moro oranges, renowned for their export potential, demand meticulous attention during transportation and storage. The application of carnauba wax (1%) on the Moro oranges resulted in a reduced postharvest loss. Furthermore, a positive and significant correlation was discovered between the total phenol content and critical parameters such as antioxidant activity, vitamin C content, and total carbohydrate content. Various authors underscore the significance of carnauba wax application on oranges and persimmons as a post-harvest preservation method. This method, when integrated with other preservation strategies, emerges as a notable approach for maintaining fruit quality during the export process^29,30^.

A comparison of mean values revealed that the highest and lowest total phenol content were observed at control (No wax) and 1% of storage, respectively (Fig. 1). Align with our findings, Germano et al.^31^ has demonstrated that storage duration has a significant impact on the total phenol content in guava fruit. Nasirifar et al. ^32^ reported that the total phenol content decreases with increasing storage time, with a 10–20% reduction in total phenol content observed after 6 months of storage. Nazoori et al.^5^ attributed the decrease in phenolic compounds during storage to the senescence process.

One of the most notable changes in Moro oranges during storage is the alteration in skin color^33^. An increase in chroma value indicates a brighter and more transparent color or, in other words, higher color quality^34^. The chroma value of the samples was higher after 40 days of storage, indicating better preservation of skin color quality compared to the control (uncoated) group (Fig. 2A–C). However, skin color, chroma, and hue values decreased after day 40 (Fig. 2A–C), a trend similarly described by Braga et al.^35^ in fruit covered with chitosan-based edible coatings containing mint essential oil. Differences in skin color, chroma, and hue values likely result from carotenoid synthesis during ripening as reported by Jing et al.^36^. Nonetheless, the higher concentration of chitosan-based edible coatings (above 18%) led to delayed color changes^37^, possibly due to reduced gas exchange induced by the coating, resulting in altered enzymatic and chemical reactions involved in chlorophyll degradation and/or pigment synthesis^38^.

The application of carnauba wax coating effectively preserves the fruit peel’s lightness (L), chroma, and hue, ensuring the visual quality remains intact while preventing both weight loss and moisture loss up to a 1% concentration level. Over the 40-day storage period, there was a notable increase in acceptance, flavor, taste, and favorable results in the panel test. Biochemical analysis revealed that a 1% carnauba wax level led to a 6% increase in vitamin C content (compared to the no-wax treatment) and a 30% increase when using a 0.5% wax concentration.

In this study, apart from measuring and assessing some of the physicochemical properties using laboratory methods, the sensory and taste aspects of the fruits were tested. Some authors have reported a correlation between post-harvest treatments and the sensory quality of fruits^39–41^. The highest scores for the panel test and overall acceptability were associated with the carnauba treatment (0.5%) on day 40 of storage.Some authors have reported a correlation between post-harvest treatments and the sensory quality of fruits^39–41^.

Sensory tests typically aim to assess the organoleptic quality of a product in comparison to its reference sample. In the case of citrus fruits, the quantitative evaluation focuses on characteristics that define fruit quality. According to our findings, the optimal fruit quality should be characterized by both chemical and sensory parameters, which were influenced by the wax coating, despite some level of decline in quality occurring during the 40 and 80 days of storage. Typically, the accumulation of ethanol and ethyl acetate compounds is used as an indicator of off-flavors in fruit during storage^42^. This was more pronounced in fruits treated with wax during the 40-day storage period compared to other treatments, although it remained within acceptable limits. The taste of fruits is primarily determined by the sugar-to-acid ratio, volatile compounds, and their synergistic effects^40^. While the flavor and taste of fruits can be altered by thermal treatments, in this study, due to changes in acidity and soluble solids being independent of the treatment type, the sensory evaluation of fruits was influenced by the prolonged storage period under the impact of the carnauba wax treatment. These findings are consistent with those reported by Njombolwana et al.^43^.

Fruits coated with carnauba wax exhibited elevated levels of total sugar content throughout storage (Fig. 3). This increase in total sugar content may be attributed to reduced consumption of specific organic acids and their conversion to carbohydrates in these fruits during storage, likely due to delayed ripening progression^44^.

Titratable acidity (TA) stands out as one of the most crucial internal quality parameters in citrus fruits, naturally undergoing changes during the stages of ripening and storage^45^. The reduction in TA during fruit ripening is a consequence of the organic acids transforming into carbohydrates. However, fruit coatings prove effective in curbing the respiratory process, thus decelerating the conversion of organic acids. This is due to the fact that organic acids serve as a substrate for respiratory pathways^46^. As a result, these coatings demonstrate efficacy in preserving citrus TA over a 40-day storage period, in agreement with previous studies^47,48^. The highest fruit acidity levels in oranges were observed in fruits treated with a 1.5% carnauba wax concentration, after 80 days of storage, also lowest total acidity (TA) was recorded in fruits treated with carnauba wax (0.5%) (Fig. 7). Biolatto et al.^49^ found that TA levels remained relatively stable during the initial stages of storage but declined towards the end of the storage period in grapefruit. This reduction in TA during storage can be attributed to the utilization of organic acids in respiration pathways as an energy source for various metabolic processes^50^. Previous studies have also documented a decline in the quality parameters of harvested fruits during storage periods^51–53^ reported a decrease in TA in citrus fruits during extended storage.

In the case of Moro oranges, vitamin C concentration initially decreased, then increased after 40 days of storage, and subsequently decreased again at the end of the 80-day storage period. In this study, the increase in vitamin C content may be attributed to synthesis or moisture loss. Similarly, García-Betanzos et al.^54^ reported the influence of storage duration and temperature on vitamin C levels in fruits. The decline in vitamin C content during storage has been observed in various fruits, including strawberries ^55^, oranges^56^, and pears^57^, and is closely linked to the fruit variety.

The decline in fruit quality, manifested as surface wrinkling and diminished juiciness, is primarily attributed to fruit moisture loss, resulting in weight loss^58^. Fruit weight loss is subject to the influence of various storage conditions in cold storage facilities, including factors such as temperature, humidity, packaging methods, and pretreatment techniques^59^. Each of these factors can have a positive, negative, or neutral effect on the weight loss process. These results are in agreement with the observations of Strano et al.^60^, who reported that storage duration plays a crucial role in citrus fruit weight reduction, which is primarily due to moisture loss. In addition, Motamedi et al.^61^ reported significant differences in fruit weight reduction during storage among different citrus species. Their research found that these differences were related to the varying thickness of postharvest coatings or protective coverings on the fruit surface, such as the natural waxes on the cuticle, which act as a barrier to water loss. Previous studies have indicated that applying carnauba wax to fresh eggplants^62^ and Mandarin fruits^63^ contributes to mitigating weight loss in the treated or coated fruits.

As the storage duration increased, there was a decline in fruit peel firmness. This aligns with the findings of Meighani et al.^64^. Our findings suggest that carnauba wax treatment can play a role in preserving the internal tissue firmness of orange fruits. Fruit firmness is a critical factor for determining both fruit freshness and consumer acceptance. While the firmness of both coated and uncoated fruits experiences a decline during storage, particularly over the 80-day duration, the coated fruits exhibit higher firmness retention compared to the control samples or untreated fruits. This firmness preservation is likely attributable to the reduction in enzymatic degradation facilitated by the coating, which restricts oxygen availability. The reduction in fruit firmness is also associated with the loss of tissue moisture and turgor pressure^65^. The retention of firmness in coated fruits can be attributed to a decrease in microbial decay, a slower respiration rate, and a modified fruit shape. This has been observed in fruits such as wax-coated plums^66^, guavas^67^, bananas^68^, and tomatoes^69^.

The authors showed that untreated batches stored for 40 and 80 days exhibited decay symptoms. In contrast, oranges treated with the coating maintained their firmness and had a significantly lower weight loss of 27% compared to untreated samples. Carnauba wax, extracted from the leaves of the Copernicia prunifera palm and often dubbed the “queen of waxes”^41^, plays a crucial role in the post-harvest citrus fruit industry. The wax acts as a protective shield for oranges throughout the storage period. The application of carnauba wax on Valencia oranges and grapefruits has proven effective in reducing fruit decay^70^. The use of carnauba wax, in combination with beeswax, has been shown to prolong the shelf life of oranges during storage by mitigating respiration^71^. Employing carnauba wax during extended storage periods, especially at lower temperatures, limited respiration, reduced moisture loss, and maintained the fruit’s firmness^32^.

A number of studies have also reported significant reductions in decay when wax-based coatings were applied to various fruits. For example, the application of carnauba wax coating led to a 4% reduction in brown rot and a 9% reduction in rhizopus rot^72^. Moreover, the decay of *Zizyphus mauritiana* fruits was reduced with the application of carnauba wax coating^73^. The coating with carnauba wax, due to its strong antimicrobial properties, led to a significant reduction in the decay rate of the coated fruit.

When compared to untreated oranges, those with the 1% carnauba wax coating exhibited a reduced level of MDA during storage. Moreover, this coating contributed to an increase in total sugars, while the total acid levels remained unchanged at the 1% concentration. As a result, the application of carnauba wax coating emerges as a potentially effective strategy for prolonging the shelf life of oranges.

Carnauba treatment, storage duration, and their interaction significantly affected this parameter. As the storage period extended to 40 days, there was an increase in fruit anthocyanin levels. These findings resonate with the research of Paolo et al.^74^, who noted a similar uptick in anthocyanin levels in blood oranges during cold storage. The synthesis of anthocyanins during postharvest cold storage varies depending on the orange variety, with some displaying higher levels than others^40^. The synthesis of anthocyanins in blood oranges after harvest is intricately linked to the activity of enzymes like phenylalanine ammonia-lyase (PAL), which experiences an upsurge during storage^75^.

The reduced antioxidant capacity after 80 days of storage may be attributed to limited anthocyanin synthesis. Previous studies have consistently reported reductions in phenolic compounds^76^, antioxidant respose^77^, and vitamin C^78^ levels in oranges, mandarins, and grapefruits during storage. Similarly, prolonged storage of citrus fruits has been associated with decreased phenolic compounds and vitamin C levels. Tavarini et al.^79^ noted that the antioxidant activity of kiwifruit was highest at harvest but decreased during extended cold storage. Candir et al.^80^ reported a decrease in antioxidant capacity during pomegranate storage. The initial rise in antioxidant activity during the storage period in citrus fruits was also documented by Supapvanich et al.^81^. Policegoudra et al.^82^ observed that the antioxidant capacity of mangoes remained stable initially but declined with prolonged storage. Asanda et al.^56^ highlighted an increase in antioxidant activity during the cold storage of oranges in all varieties, primarily attributed to phenolic compounds and vitamin C, which presents an interesting contrast to the findings of our study.

Correlation coefficients are beneficial for measuring the interplay between two parameters and illuminating the extent and direction of their relationship. In the field of breeding, their application is of great importance^83^. The correlation coefficient showed a simultaneous significant increase in antioxidant activity 40 and 80 days after storage, which was accompanied by an increase in total phenol content. Similarly, both chroma and hue showed a significant increase associated with the increased skin radiance. These results highlight that most parameters were strongly positively correlated, meaning that improvements in one trait can positively and beneficially affect the others. Moreover, a significant and positive correlation between antioxidant activity and MDA levels in different citrus varieties has already been documented in the literature^84–86^. Cluster analysis serves as a statistical technique, for grouping the traits or the differentiation of various populations based on a variety of characteristics^87–89^. In our experiment, fruit weight reduction, formed individual clusters (Fig. 15).

## Conclusions

In this study, edible carnauba wax was used to extend the shelf life of orange fruit. While wax coating increased the visual appeal of fruit, its impact on nutritional value during postharvest storage should not be overlooked. Carnauba wax is a practical choice due to its cost effectiveness and domestic production. Anthocyanin content in the fruit increased regardless of storage time. The naturally strong antibacterial properties of carnauba wax helped to maintain the weight, TA, and firmness of the coated or treated fruits over a storage period of 40 days. Under storage conditions, the carnauba wax coating proved to be highly effective in minimizing fruit decay and extended the shelf life of the fruits by up to 80 days at concentrations of 1% and 0.5%. The results of this study suggest that active wax-based coatings are a practical and sustainable alternative for preserving and extending the shelf life of citrus fruit during conventional cold storage.

## Data Availability

All the data generated/ analyzed during the study are available with the corresponding author on reasonable request.
